# The “Big Three” in biocompatibility testing of medical devices: implementation of alternatives to animal experimentation—are we there yet?

**DOI:** 10.3389/ftox.2023.1337468

**Published:** 2024-01-08

**Authors:** Helena Kanďárová, Peter Pôbiš

**Affiliations:** Institute of Experimental Pharmacology and Toxicology (IEPT), Centre of Experimental Medicine (CEM), Slovak Academy of Sciences (SAS), Bratislava, Slovakia

**Keywords:** medical device, cytotoxicity, irritation, sensitisation, the international organization for standardization (ISO), ISO 10993, *in vitro*

## Abstract

Biocompatibility testing ensures the safety of medical devices by assessing their compatibility with biological systems and their potential to cause harm or adverse reactions. Thus, it is a critical part of the overall safety evaluation process for medical devices. Three primary types of biocompatibility tests—cytotoxicity, irritation, and sensitisation assessment—are standard for nearly all medical devices. However, additional biocompatibility tests, such as genotoxicity, systemic toxicity, hemocompatibility, and implantation studies, may also be necessary, depending on the device’s nature and intended use. The testing is partly conducted *in vitro*, but the industry still heavily relies on animal experiments. Compared to other industrial sectors, implementing alternatives in medical device biocompatibility testing has been notably slower. This delay can be attributed to the absence of specific validation processes tailored to medical devices and the resulting hesitation regarding the predictive capacity of these alternative methods despite their successful applications in other domains. This review focuses on the progress and obstacles to implementing new approach methodologies in the areas of cytotoxicity, irritation and sensitisation testing of medical devices. While challenges persist in adopting these innovative methods, the trend towards embracing alternatives remains robust. This trend is driven by technological advancements, ethical considerations, and growing industrial interest and support, all collectively contributing to advancing safer and more effective medical devices.

## 1 Introduction

Biocompatibility testing is a pivotal element within the medical device development and regulatory approval processes, ensuring their safety and compatibility when interacting with biological systems. Central to this testing are the “Big Three” assessments, namely, cytotoxicity, irritation, and sensitisation testing, which must be performed for almost all medical devices being introduced to the market. Depending on the type of the medical device and its intended use, additional tests may to be carried out; still, the “Big Three” remain the cornerstone of the biocompatibility assessment.

Throughout history, animals have been used in medical research to ascertain the safety and efficacy of pharmaceutical products and medical devices prior to human use. Nevertheless, animal testing gives rise to numerous ethical dilemmas and remains a topic of public contention. While these established tests play a crucial role in safety assessment, the medical device industry also recognises the potential of alternative approaches that could accelerate and streamline the safety testing process.

In 2010, the European Parliament endorsed Directive 2010/63/EU which aims to protect animals used in scientific research (Directive 2010/63/EU, 2010). The directive requires the integration of the “3Rs” principles (Replacement, Reduction, and Refinement) and establishing precise welfare standards for animals across all phases of product development, manufacturing, and testing. (EU 2017/745, 2017), governing medical devices, follows and enforces compliance with Directive 2010/63/EU.

According to the ISO 10993-1:2018 standard, animal testing is only justified when existing scientific data and *in vitro* studies fail to provide adequate information for a comprehensive assessment of the safety of a medical device (ISO 10993-1:2018, 2018). Furthermore, ISO 10993-2:2022, outlines the minimum requirements necessary to ensure and substantiate the ethical treatment and welfare of animals when conducting mandatory animal testing (ISO 10993-2:2022, 2022).

Unfortunately, compared to other industrial sectors, the integration of alternative approaches in medical device biocompatibility testing has been notably slow. This delay can be attributed to the absence of specialised validation processes tailored to medical devices and the consequent regulatory scepticism and hesitation surrounding the predictive capacity of these alternatives despite their successful applications in other domains.

This review delves into the “Big Three” biocompatibility tests for medical devices and explores the progress and challenges of implementingalternativemethods in cytotoxicity, irritation, and sensitisation testing. We aim to shed light on the reasons behind this cautious approach and the potential avenues for accelerating the adoption of alternative biocompatibility testing techniques in the medical device industry.

## 2 Regulatory frameworks

Compliance with national and international biocompatibility testing requirements is essential for regulatory approval and the safe use of medical devices in hospitals, healthcare settings or by naïve end-users. Regulations related to medical devices and biocompatibility testing vary by country or region. Still, some common international standards and regulations are widely recognised and followed by industry. Key regulations and standards that deal with biocompatibility testing of medical devices include:

*ISO 10993 Series:* International Organization for Standardization Technical Committee 194 (ISO/TC 194) developed the globally harmonized ISO 10993 standards, which provide guidance and requirements for evaluating the biocompatibility of medical devices. ISO 10993 standards cover various aspects of biocompatibility testing, including cytotoxicity, sensitisation, irritation, genotoxicity, and more. Manufacturers often use these standards as a reference for conducting biocompatibility testing and assessing the safety of their medical devices.

In some areas, the OECD test guidelines (TGs) can be used. OECD TGs are comprehensive set of protocols primarily designed to assess the safety of chemical substances and mixtures, and they play a significant role in the evaluation of certain aspects of medical devices. While these guidelines offer a standardized approach for safety assessments, medical devices often require additional, more specific evaluations as per ISO series.

*Food and Drug Administration (FDA):* Is the U.S. regulatory authority responsible for overseeing medical devices. The FDA has specific regulations related to biocompatibility testing, including guidance documents and standards that align with ISO 10993; however, it does not fully recognize all ISO 10993 standards. Manufacturers must provide biocompatibility data with their regulatory submissions for FDA clearance or approval.

*European Medical Device Regulation (MDR):* Is a comprehensive regulatory framework governing medical devices in the EU, including requirements related to biocompatibility testing. Manufacturers must comply with this regulation to obtain CE marks for their devices. The MDR references ISO 10993 standards and outlines the expectations for biocompatibility assessment.

*Pharmaceuticals and Medical Devices Agency (PMDA):* Japan’s PMDA oversees the regulation of medical devices. PMDA’s requirements for biocompatibility testing are aligned with international standards and guidelines. Manufacturers seeking approval for their devices in Japan must adhere to these regulations and provide biocompatibility data.

*Health Canada Regulations:* Health Canada regulates medical devices in Canada. Manufacturers must meet the requirements outlined in the Medical Devices Regulations (MDR) and submit biocompatibility data for device approval. These regulations align with international standards, including ISO 10993.

Many other countries have their own regulatory authorities and requirements for medical devices and biocompatibility testing. Manufacturers should consult the relevant regulatory authorities in each country where they intend to market their devices to ensure compliance with local regulations.

Medical device manufacturers must know and comply with the specific regulations and standards applicable to their products. Manufacturers typically work closely with regulatory experts and contract research organisations (CROs) specialising in biocompatibility testing to meet these requirements. Regrettably, despite repeated efforts, the absence of harmonization within the field has resulted in confusion and a certain level of ambiguity in testing prerequisites and ultimate assessments.”

## 3 The “Big Three” in biocompatibility testing and ISO *in vitro* approaches

The “Big Three” refers to cytotoxicity, irritation and sensitisation testing. Testing of these three biological effects is required on most medical devices regardless of category, patient contact, and duration of use (Lyons, 2022).

Medical devices are predominantly tested as extracts, prepared by immersing the device or its components in an appropriate extraction solvent such as physiological saline, vegetable oil, or cell culture medium, under specified conditions. This extraction process is a standard method for assessing the biocompatibility of medical devices by evaluating the potential release of substances that could interact with biological systems. Details on extract preparation are given in ISO 10993-12 (ISO 10993-12:2021, 2021). Although the ISO standards provide guidance on test sample selection and preparation, experimental controls, reference materials, and extraction, CRO’s conducting the testing may interpret specific recommendations differently, which may lead to variability in results.

### 3.1 Cytotoxicity testing

The primary purpose of cytotoxicity testing is to assess whether a medical device’s materials and components can potentially cause harm to living cells. This testing helps determine whether the device or its extracts are safe for use in contact with biological systems, such as human or animal tissues and cells. It is crucial to ensure that the device does not harm cells when it contacts the body, as this can lead to adverse effects and complications. Cytotoxicity testing, as specified in ISO 10993-5:2009 is essential for biocompatibility assessment of medical devices. The standard provides guidance and requirements for evaluating the cytotoxic potential of materials used in medical devices (ISO 10993-5:2009, 2009).

#### 3.1.1 *In vitro* test methods and protocols

ISO 10993-5 provides specific test methods and protocols for conducting cytotoxicity testing. These methods typically involve exposing cultured mammalian cells to extracts of the medical device or its materials for approximately 24 h. Commonly used cell lines for cytotoxicity testing include Balb 3T3 (fibroblasts), L929 (fibroblasts) and Vero (kidney-derived epithelial cells). Cytotoxicity testing evaluates various endpoints to assess cell viability and adverse cellular reactions. The primary endpoints include:

• Cell viability: This measures the extent to which cells exposed to the device extracts survive and proliferate compared to control cells.
• Morphological changes: Any changes in cell shape or structure are noted.
• Cell detachment: The degree of cell detachment from the culture substrate is assessed.
• Cell lysis: The presence of cellular debris or cell membrane damage is evaluated.

Based on these endpoints, cytotoxicity is typically categorized as non-cytotoxic, mildly cytotoxic, moderately cytotoxic, or highly cytotoxic. The following methods are commonly used to determine quantitative cell viability for these categories: MTT (3-(4,5-dimethylthiazol- 2-yl)-2,5-diphenyl-2Htetrazolium bromide), XTT (2,3-bis-(2-methoxy-4-nitro-5-sulfophenyl)- 2H-tetrazolium-5-carboxanilide), and neutral red uptake. Other less frequently used methods are the Bradford protein, Chrystal violet, Resazurin dye, and Trypan blue assays (Gruber and Nickel, 2023).

#### 3.1.2 Acceptance criteria

ISO 10993-5 does not define specific acceptance criteria for cytotoxicity testing; however, its Annex V provides guidance for data interpretation, where protocols are detailed. This ISO standard emphasizes that the acceptance criteria should be defined based on the nature of the medical device, its intended use, and potential patient exposure. If cytotoxicity is observed, further testing should be conducted to better understand the influence of the test conditions on the result. Any cytotoxic effect can be of concern; however, the medical device cannot necessarily be determined unsuitable for a given clinical application based solely on cytotoxicity data. On the other hand, 70% cell survival (cell viability) and above can be seen as a positive sign, especially when testing neat extract.

#### 3.1.3 Relevance to regulatory compliance

According to ISO 10993-5, cytotoxicity testing is a fundamental component of the biological evaluation of medical devices. It is the primary test required by regulatory authorities such as the US FDA, European Medicines Agency (EMA), PMDA, and other national agencies. Manufacturers use the results of cytotoxicity testing to support regulatory submissions and demonstrate the safety of their devices.

While ISO 10993-5 does include *in vitro* cytotoxicity testing as a central component of biocompatibility evaluation, it is part of a broader framework that considers various aspects of biocompatibility, including other *in vitro* and *in vivo* tests, as well as risk assessment. The specific tests and evaluations conducted for a given medical device will depend on its characteristics and intended use to ensure its safety and compatibility with biological systems.

### 3.2 Irritation testing

The ISO 10993-23:2021 standard provides updated guidance for assessing the skin irritation potential of medical devices (ISO 10993-23:2021, 2021). A key aspect of this standard is its strong support for the *in vitro* reconstructed human epidermis (RhE) assay as the preferred method over traditional *in vivo* animal tests. This shift aligns with ethical efforts to reduce animal testing and reflects a combined industrial and regulatory commitment to advancing biocompatibility evaluation methods. Based on the old ISO 10993-10:2010 standard, which concerned skin irritation and sensitisation, ISO 10993-23:2021, was developed thanks to over a decade of collaborative effort by industry partners validating the RhE assays for medical devices.

This collaboration led to the ISO/TC 194s decision to update and separate these two endpoints into distinct standards - ISO 10993-23:2021 for irritation and ISO 10993-10:2021 for sensitization testing needs of the medical device industry (ISO 10993-23:2021, 2021; ISO 10993-10:2021, 2021).

#### 3.2.1 ISO 10993-23:2021’s *in vitro* test methods

*In vitro* test methods and protocols within ISO 10993-23:2021 describe specific procedures for skin irritation assessments using advanced models (ISO 10993-23:2021, 2021). The standard endorses validated RhE models EpiDerm and SkinEthic RHE. These three-dimensional, cultured skin models closely replicate human epidermal tissue’s barrier properties and structure, making them highly relevant for irritation and intracutaneous testing. Their applications extend to various regulatory domains, including skin corrosion, irritation, and phototoxicity evaluations for chemicals, cosmetics, and drugs, as delineated in OECD TG 431, 439, and 498 and ICH S10 (EMA, 2014; OECD, 2019; OECD, 2021; OECD, 2023).

The potential for RhE models to replace traditional animal testing was highlighted by Casas et al. (2013) which demonstrated their ability to identify chemical irritants in medical device extracts. This work spurred ISO/TC 194 to encourage further development and validation of these methods. A key initiative was a global round robin study designed to assess the RhE models’ predictive capability in identifying irritating properties of medical device extracts. For this study, three organisations provided positive and negative samples of medical device polymers; in addition, human patch tests were conducted alongside for comparative analysis.

The comprehensive results of this study, conducted by 23 laboratories worldwide between 2015 and 2017, along with other related scientific findings, were published in a special medical device issue of Toxicology *In vitro* (2018). These results led to the creation of ISO 10993- 23:2021 by ISO/TC 194s Working Group 8 for Irritation and Sensitization (De Jong et al., 2018a; Kandarova et al., 2018a; De Jong et al., 2018b; Coleman et al., 2018; Pellevoisin et al., 2018).

The validated testing protocols of ISO 10993-23:2021 involve an 18–24 h exposure of the RhE models to medical device extracts, followed by assessments of cellular damage and inflammatory responses. These assessments are typically conducted using cell viability assays, such as the MTT test, and cytokine release profiling, ensuring a robust and comprehensive evaluation of a material’s irritation potential (ISO 10993-23:2021, 2021).

#### 3.2.2 Acceptance criteria

The assessment of tissue viability via cytotoxicity testing plays a pivotal role in determining the irritation potential of medical device extracts or topically applied formulations. The primary indicator of irritation is the reduced viability of cells within the RhE model. A decrease in cell viability below 50% is considered a sign of irritation. However, a significant decrease in viability, when coupled with a notable increase in interleukin-1 alpha (IL-1α), can also indicate tissue inflammation.

In addition, the reliability of RhE models was further confirmed by parallel testing conducted with human volunteers and comparative analysis with existing rabbit data which demonstrated that RhE models closely mirrored the predictions of traditional rabbit intracutaneous skin tests (Kandarova et al., 2018b). This agreement underscored the high sensitivity and predictive accuracy of RhE models in assessing the irritation potential of medical devices, making them a robust alternative in biocompatibility testing.

#### 3.2.3 Relevance to regulatory compliance

For the vast majority of medical device manufacturers, complying with ISO 10993-23:2021 has become critical for achieving regulatory compliance in major international markets. Consequently, this new standard has been rapidly adopted CROs. However, the regulatory landscape is not uniform. While Europe and Asia have embraced *in vitro* testing, the US FDA has yet to recognize the *in vitro* testing sections of the standard and still requires irritation data from rabbits (FDA, 2021).

The medical device industry is working with FDA to satisfy its request for dual data from the *in vitro* RhE assays and *in vivo* rabbit tests, along with data from previous validation studies for chemicals and cosmetics (De Jong et al., 2020).

This divergence in regulatory requirements between Europe, Asia and the U.S. presents a significant challenge for the medical device industry, creating a situation of dual testing. Such discrepancies not only complicate the global compliance process, but also have significant impacts on the costs and efficiency of testing. This situation underscores the need for global harmonisation in medical device testing standards, which is crucial for streamlining the approval process and reducing unnecessary financial and procedural burdens.

### 3.3 Sensitization testing

Sensitisation testing is critical in evaluating medical devices and their materials for potential allergic or hypersensitivity reactions. This testing aims to determine if a device can sensitise the immune system, leading to allergic responses upon subsequent exposures. The standard animal-based sensitisation tests are the Guinea Pig Maximization Test (GPMT), Buehler assay, and murine Local Lymph Node Assay (LLNA). Of these, the GPMT is recognised as the most sensitive method.

Despite significant advancements in the chemical industry, and incorporation of various methods into OECD Test Guidelines based on knowledge of key events leading to sensitisation, the medical device industry has not yet incorporated these *in vitro* and in chemico methods into the ISO 10993 standards, but still relies on animal testing for decision-making (Kerecman Mayers et al., 2017).

ISO 10993-10:2021 acknowledges that alternative approaches for neat chemicals have been developed, utilising a combination of assays to identify skin sensitisers (ISO 10993-10:2021, 2021). These methods are included in OECD Test Guidelines (TG 442C, TG 442D, and TG 442E) or are part of the ongoing OECD test guideline evaluation program. An overview of the methods can be found in Annex C of the ISO 10993-10:2021 (Table 1). However, the applicability of these alternative approaches for medical devices remains uncertain, and validation studies are necessary to demonstrate the reliability and relevance of these tests for the medical device industry. This issue is being addressed by ISO/TC 194s Working Group 8 that recently published ISO/TS 11796:2023, which provides detailed guidance on conducting an *in vitro* sensitisation validation study for medical devices. In 2024, Working Group 8 plans to begin preliminary work on a global round robin study of *in vitro* sensitization methods (ISO/TS 11796:2023, 2023).

**TABLE 1:** Overview of in chemico and *in vitro* tests for skin sensitisation testing included in the informative Annex C of the (ISO 10993-10:2021, 2021).

No.	Method name	OECD TG	Key event	Principle of the test
1	DPRA	OECD TG 442C	Key event 1: Molecular initiating event of colvalent protein binding	Cysteine and lysine peptide percent depletion values are determined and used in a prediction model that assigns the test chemical to one of four reactivity classes that categorize them as skin sensitizers or non-sensitizers
2	KeratinoSens™	OECD TG 442D	Key event 2: Activation of epidermal keratinocytes	The KeratinoSens™ cell line contains the luciferase gene under the transcriptional control of a constitutive promoter fused with the ARE element. The luciferase signal indicates the activation of endogenous Nrf2 dependent genes by electrophilic skin sensitizers. Luciferase gene induction is determined quantitatively by measuring luminescence produced by light generating luciferase substrates. Test chemicals are considered skin sensitizers if they induce a statistically significant increase in luciferase activity (i.e., a 50% increase), below a concentration which does not cause a significant reduction in cell viability
3	LuSens	OECD TG 442D	Key Event 2: Activation of epidermal keratinocytes	The LuSens transgenic cell line contains a luciferase reporter gene under the transcriptional control of a promoter fused with the ARE element. The luciferase signal reflects the activation by electrophiles of endogenous Nrf2 dependent genes. Luciferase gene induction is quantitatively determined by luminescence measurement of light producing luciferase substrates, as an indicator of the activity of the Nrf2 transcription factor in cells following exposure to electrophilic skin sensitizers
4	SENS-IS	Project 4.107: New TG: Toxicogenomic analysis on 3D reconstituted epidermis for measuring skin sensitization potency — the SENS-IS assay	Key Event 2: Activation of epidermal keratinocytes	Gene expression of two groups of genes is measured: one group (REDOX group) includes a selection of 17 genes that have an antioxidant responsive element in their promoter and monitor the redox protective signals induced through the interaction of skin sensitizers binding to cysteine amino acids of the Keap1-NRF2 complex. The second group (SENS-IS group) includes a selection of 21 genes involved in inflammation, danger signals and cell migration to address the complex cascade of events leading to activation of DCs by a skin sensitizing chemical
5	IL-18 RhE assay	None at this time	Key Event 2: Activation of epidermal keratinocytes	Reconstructed human epidermis models SkinEthic™ RHE (EPISKIN), VUmc-EE, Ep-iCS® (CellSystems), and EpiDerm™ (MatTek) are used to determine the IL-18 content, as marker of sensitisation. At the end of chemical exposure, the epidermises are subjected to the cell viability assay and the maintenance media is analysed for the IL-18 content by ELISA testing
6	EpiSensA	None at this time	Key Event 2: Activation of epidermal keratinocytes	This assay is based on the induction of multiple marker genes (ATF3, IL-8, DNAJB4 and GCLM) related to two keratinocyte responses (inflammatory or cytoprotective) in the induction of skin sensitization. The mechanistic relevance of the marker genes has been confirmed by focusing on key molecules that regulate keratinocyte responses *in vitro* (P2X7 for inflammatory and Nrf2 for cytoprotective responses). The upregulation of ATF3 and IL-8, or DNAJB4 and GCLM induced by 2,4-dinitrochlorobenzene in human keratinocytes is significantly suppressed by a P2X7 specific antagonist KN-62, or by Nrf2 siRNA, respectively, which supports the mechanistic relevance of the marker genes
7	SenCeeTox**®**	None at this time	Key event 2: Activation of epidermal keratinocytes	The expression of eight Nrf2/ARE, one AhR/XRE and two Nrf1/MRE controlled gene are measured by quantitative reverse transcription- polymerase chain reaction (qRT-PCR). The fold-induction at each exposure concentration is combined with reactivity and cytotoxicity data to determine the sensitization potential
8	h-CLAT	OECD TG 442E	Key event 3: Activation of epidermal dendritic cells	The h-CLAT assay measures changes in the expression of CD86 and CD54 cell surface markers on THP-1 cells after exposure to the test chemical for 24 h. These surface molecules are typical markers of monocytic THP-1 activation and can imitate dendritic cell activation, which plays an important role in T-cell priming. Changes in surface marker expression are measured by fluorescence-based flow cytometry. The relative fluorescence of the surface markers compared to control vehicles are determined and used to differentiate between skin sensitizers and non-sensitizers
9	U-SENS™	OECD TG 442E	Key event 3: Activation of epidermal dendritic cells	CD86 is known to be a co-stimulatory molecule that can mimic monocytic activation, which plays a critical role in T-cell priming. The changes of CD86 cell surface marker expression are measured by flow cytometry following cell staining typically with fluorescein isothiocyanate (FITC)-labelled antibodies. Cytotoxicity measurement is also conducted concurrently to assess whether upregulation of CD86 cell surface marker expression occurs at sub-cytotoxic concentrations. The stimulation index (SI) of CD86 cell surface marker compared to solvent/vehicle control is calculated and used in the prediction model, to support the discrimination between skin sensitizers and non-sensitizers
10	IL-8 Luc assay	OECD TG 442E	Key event 3: Activation of epidermal dendritic cells	The IL-8 Luc assay uses a THP-1-derived IL-8 reporter cell line, THP-G8, that harbours the stable luciferase orange (SLO) and stable luciferase red (SLR) luciferase genes under the control of the IL-8 and glyceraldehyde 3- phosphate dehydrogenase (GAPDH) promoters, respectively. This allows quantitative measurement of luciferase gene induction by detecting luminescence from well-established light producing luciferase substrates as an indicator of the activity of the IL-8 and GAPDH in cells following exposure to skin sensitizing chemicals
11	GARD™ Genomic allergen rapid detection™	Project 4.106: New TG: GARD™skin test: An *in vitro* method for identification of skin sensitizers based on a genomic interpretation of the impact of chemicals on human dendritic cell-like cells (AOP key event 3)[	Key event 3: Activation of dendritic cells	GARD™skin measures the gene expression of 200 genes induced in SenzaCells™ in response to chemical exposure. The 200 genes, referred to as GARD prediction signature (GPS) includes biomarkers for dendritic cell activation and maturation (KE3), several danger signal pathways and pattern recognition receptors (KE2), and antigen presenting molecules and cell proliferation pathways (KE4)

## 4 Obstacles to implementing additional *in vitro* tests for other toxicity endpoints

A range of *in vitro*, *in silico*, and in chemico assays have been developed for assessing biological endpoints, including skin and eye irritation, as well as skin sensitisation, for cosmetics, pharmaceuticals, and chemical substances. However, their validation and acceptance for medical device use remain pending because the medical device testing field has been reluctant to adopt new approach methodologies. A review of the reasons for the slow validation and implementation of *in vitro* testing methods is presented below.

### 4.1 Technical difficulties of testing materials by *in vitro* methods

*Degradation of medical devices:* Over time, both chemical and mechanical degradation can lead to delayed cytotoxic or inflammatory responses. This process presents a significant challenge for current *in vitro* testing methods which are typically designed for short-term assessment. *In vitro* assays may not adequately simulate prolonged, repeated exposure, and the cumulative effects that medical devices experience under real-life conditions. Capturing these long-term and repeated toxicity effects *in vitro* is a complex task.

One potential avenue to address this challenge is using microfluidic systems combined with advanced cell culture models. These systems have the potential to culture cells over extended periods, thereby providing a more realistic simulation of long-term device usage and its effects. Furthermore, addressing the issue of material degradation–whether mechanical or chemical–is essentially an engineering challenge. It requires the integration of interdisciplinary teams in the design of testing methods. By involving experts from various fields, including material science, bioengineering, and toxicology, more comprehensive and predictive *in vitro* models can be developed. These models would assess immediate cytotoxic effects and evaluate the long-term biocompatibility and safety of medical devices.

*Low concentration of toxic compounds:* Medical device extracts are often complex chemical mixtures, wherein harmful components might be present at low concentrations. Although trace levels can pose significant risks over long-term exposures, accurately assessing these risks in short-term *in vitro* acute toxicity tests is difficult.

*Challenges in sample preparation:* The methodology for preparing extracts from medical devices needs more standardization and harmonization. Recent studies evaluating the variability of ISO 10993-5:2009 cytotoxicity methods have highlighted the substantial impact of the extracting solution—such as medium with or without serum—on test outcomes. Even minor protocol modifications can significantly alter the predicted cytotoxicity effects (Jablonska et al., 2021).

There is a need for more comprehensive guidance on handling materials that absorb solvents, as they can alter the osmolarity of the cell culture medium, adversely affecting the cell lines. Testing poorly soluble materials in submerged cell cultures, in general, poses technical challenges and may lead to false-negative results. In addressing these issues, epithelial 3D tissue models emerge as a promising solution. These models are capable of sustaining materials extracted in both polar and non-polar solutions, offering a more versatile and potentially accurate testing framework.

The development and implementation of such advanced models could significantly enhance the reliability of cytotoxicity assessments for medical devices, particularly for those with low-level toxic components that are poorly soluble in polar vehicles. This approach would ensure a more accurate long-term safety and efficacy prediction, aligning *in vitro* testing more closely with real-world device usage scenarios.

### 4.2 Slow adaptation of existing protocols

*Protocol adjustment delays:* The medical device industry has been slow in adapting and validating existing testing protocols from other sectors to suit the unique properties of medical devices. This delay is partly due to the lack of well-characterised medical device materials that can serve as positive controls for specific toxicity endpoints.

In the past, materials that were identified as problematic were quickly removed from the market. This, however, creates a challenge for test method developers, who require access to medical device manufacturers capable of producing test R&D samples spiked with known irritants, sensitisers, or other materials of interest for effective test development.

*Limited validation expertise:* The complexity of medical device testing necessitates specialised expertise for validation projects. However, only a few CROs and medical device manufacturers possess the necessary skills and resources (financial and personal) to design and conduct such validation projects effectively, leading to bottlenecks in broader validation and consequent implementation.

Although there are test methods and models that could be included in ISO 10993-23:2021 for other endpoints (e.g., eye, oral, and vaginal irritation), validation studies have yet to be completed (ISO 10993-23:2021, 2021). Similarly, some of the OECD 442 *in vitro* and *in chemico* methods just need a interlaboratory trial with well-selected samples to prove their acceptability for medical device testing. This has not happened yet, however, as described above, ISO/TC 194 Working 8 is currently laying the groundwork for such studies.

### 4.3 Regulatory distrust and lack of public interest

*Regulatory skepticism towards alternatives:* A notable challenge in adopting alternative testing methods is the skepticism displayed by some national regulatory bodies. Even though many *in vivo* tests have never formally been validated, regulators often prefer these established methods over newer, industry-developed *in vitro* alternatives.

*Industry-regulator collaboration:* A potential solution is fostering closer cooperation between industry and regulatory authorities. This collaboration could involve industry providing more test materials and involving regulators early in the method development and validation stages, potentially transforming the current dynamics.

*Regulatory interest in comparative data:* Some regulators, such as FDA, are keen to see how *in vitro* data compares to traditional animal study results. The medical device industry, therefore, should be prepared to open its archives and conduct additional *in vitro* tests, allowing for a comprehensive comparison with historical animal data. This approach could help build trust and demonstrate alternative methods’ efficacy.

*Lack of public pressure/minimal advocacy:* Unlike other industries, the medical device sector has experienced less public pressure or animal rights groups campaigning to adopt alternative testing methods. This lack of public engagement may contribute to the slower pace of change and acceptance of non-animal testing methods in this sector.

### 4.4 Cross-sectorial harmonisation, open access to the information

*Cross-sectorial harmonisation:* The medical device industry currently grapples with challenges in harmonisation stemming from varied standards and practices among companies and across different global regions. This disparity impedes the adoption of new testing methods and risks creating inconsistencies in assessing medical device safety.

Achieving harmonisation requires a coordinated effort across various industry branches, scientific disciplines, and regulatory bodies, extending to an international level. ISO is pivotal in ensuring this global uniformity. A key strategy would involve initiating an active dialogue among national parties, akin to the OECD Mutual Acceptance of Data (MAD) process. Such a dialogue is essential for aligning standards and practices, thus facilitating a more seamless adoption of innovative testing methodologies (Kerecman Myers et al., 2017).

*Open access to information:* To keep pace with rapid technological advancements and ensure that testing methodologies accurately reflect the current state of scientific progress, the harmonisation process needs to be expedited. An essential aspect of this effort is open access to information. Transparent sharing of data, research findings, and methodological advancements is crucial for fostering collaboration, driving innovation, and ensuring that all stakeholders are informed and engaged in the harmonisation process.

### 4.5 Training of regulators along with contract research organizations

The practical training of CROs and regulatory bodies is vital in the evolving landscape of medical device testing. CROs, critical intermediaries in developing and validating medical devices, require extensive training in cutting-edge testing methodologies, regulatory compliance across various jurisdictions, and ethical testing practices. This training includes a deep understanding of *in vitro* methods, computational modelling, and the latest ISO 10993 standards. Similarly, training for regulators is equally crucial to ensure they are well-versed in the latest scientific developments and testing methodologies. This dual training approach ensures that CROs and regulators share a common understanding of the current best practices and challenges in medical devicetesting.

For regulators, training should focus on the nuances of new testing technologies, data interpretation, and the implications of these advancements on regulatory policies. This knowledge is critical for informed decision-making regarding the approval of medical devices. Additionally, the training should foster an understanding of the industry’s perspective, aiding in more collaborative and effective regulatory processes.

Collaboration is vital in these training initiatives. Joint training sessions, workshops, and seminars involving CROs and regulatory personnel can foster mutual understanding and communication. Continuous updates and refreshers on training content are essential to keep pace with the rapidly evolving field. By investing in the thorough and ongoing training of CROs and regulators, the medical device industry can more effectively bridge the gap between innovation, safety, and regulatory compliance.

## 5 Conclusion

The slow progression in validation and implementation of *in vitro* testing methods in the medical device sector is multifaceted. It is influenced by technical challenges, the inherent complexity of medical devices, regulatory hesitancy, limited advocacy for alternative methods, the specialised nature of the required testing, and a lack of industry-wide harmonisation.

The language in the ISO 10993-1:2018 standard acknowledges the possibility of a tiered approach, emphasizing the importance of *in vitro* data (ISO 10993-1:2018, 2018). However, there is a notable gap between recognition and practical application in regulatory decision-making. Nevertheless, implementing this approach comprehensively across the “Big Three” endpoints presents a substantial challenge, particularly given the unique complexities associated with sensitisation testing.

Addressing these issues requires concerted efforts by industry stakeholders, regulatory bodies, and the scientific community to advance towards more efficient, ethical, and reliable testing methodologies.
